# Progesterone Hypersensitivity Induced by Exogenous Progesterone Exposure

**DOI:** 10.7759/cureus.44776

**Published:** 2023-09-06

**Authors:** Gurnoor Dhaliwal, Jaskaran Batra, Anvitha R Ankireddypalli, Swathi Gorle, Ashok Kumar Kanugula, Jasleen Kaur

**Affiliations:** 1 Endocrinology, HealthPartners, Minneapolis, USA; 2 Internal Medicine, University of Pittsburgh Medical Center (UPMC), McKeesport, USA; 3 Endocrinology, Baptist Health Surgical and Specialty Clinic, Conway, USA; 4 Internal Medicine, Wellstar Spalding Regional Medical Center, Griffin, USA; 5 Internal Medicine, Wellstar Spalding Regional Hospital, Griffin, USA; 6 Endocrinology, Diabetes and Metabolism, HealthPartners, Minneapolis, USA

**Keywords:** progesterone, intramuscular progesterone, angioedema, urticaria, autoimmune progesterone dermatitis, progesterone hypersensitivity

## Abstract

Progesterone hypersensitivity (PH) is a rare hypersensitivity reaction to either endogenous or exogenous progesterone. There are around 200 reported cases of progesterone hypersensitivity in the medical literature. We present the case of a 31-year-old female who presented with cyclical urticaria and angioedema after exogenous progesterone exposure. Her symptoms would begin a few days before her menstrual cycle began and resolve after menstruation. She only had partial recovery of her symptoms with antihistamines, steroids, montelukast, and omalizumab. She needed treatment with oral contraceptives and had a resolution of symptoms, but subsequently developed a recurrence again. Given the rarity of this condition, the diagnosis is often delayed. This diagnosis should be considered for women of reproductive age who present with cyclic hypersensitivity or allergic symptoms.

## Introduction

Progesterone hypersensitivity (PH) is an under-recognized hypersensitivity reaction to endogenous or exogenous progesterone [1]. This condition is almost exclusively seen in women of reproductive age, with symptoms occurring during the luteal phase of the menstrual cycle [2]. The symptoms typically start three to seven days before menstruation, when the progesterone levels peak around day 21 of a 28-day menstrual cycle [3]. The symptoms can last one to three days after menstruation begins. Common symptoms include cutaneous manifestations of urticaria and angioedema. Some women can present with asthma-like symptoms or even anaphylaxis. The diagnosis of this condition is challenging and often delayed due to its rarity and heterogeneous presentation, along with variability in progesterone exposure [4]. Common sources of exogenous progesterone that can result in PH include oral contraceptives, medroxyprogesterone, intrauterine devices containing progesterone, and vaginal rings. The prevalence of this condition is expected to rise in the coming years due to the widespread use of progestins in various contraceptives and fertility treatments [1,5]. Herein, we report the case of a 31-year-old female who developed progesterone hypersensitivity after exposure to exogenous progesterone therapies.

## Case presentation

**Figure 1:**
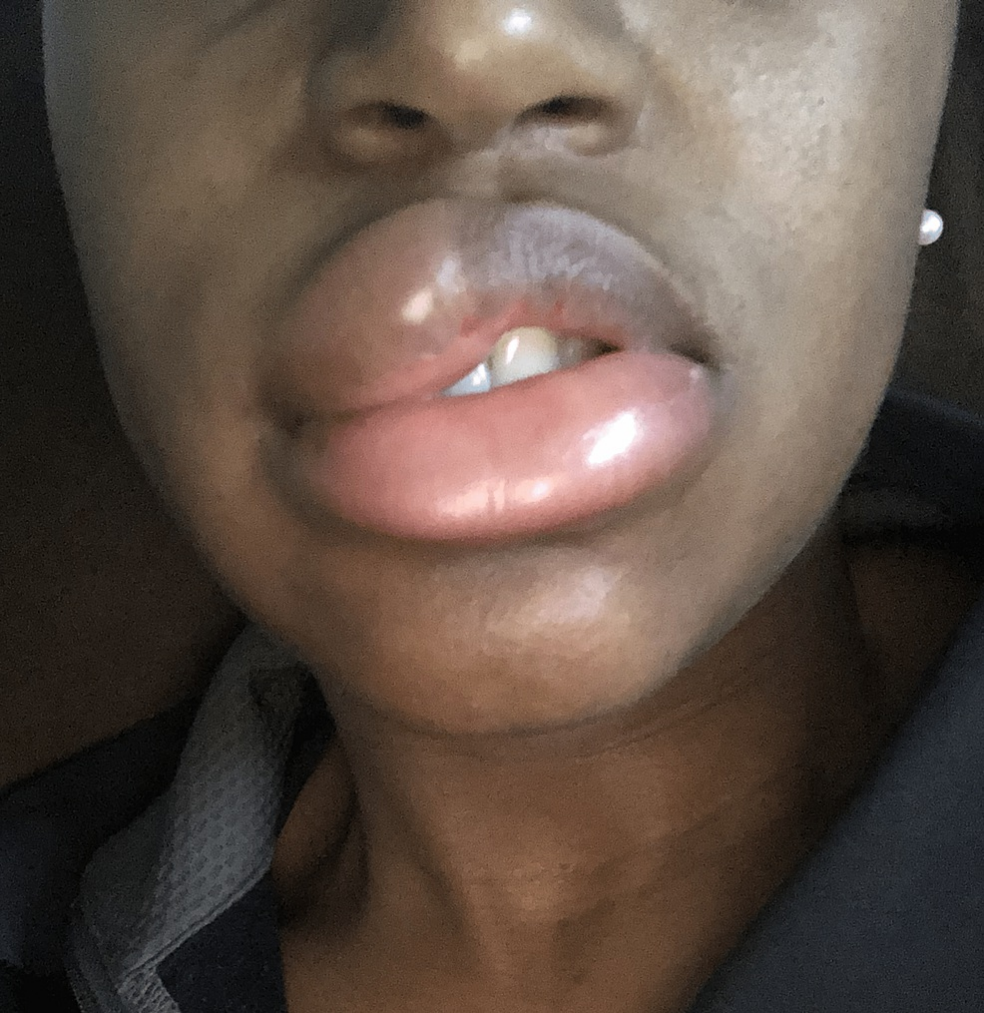
Angioedema involving the lips

**Figure 2:**
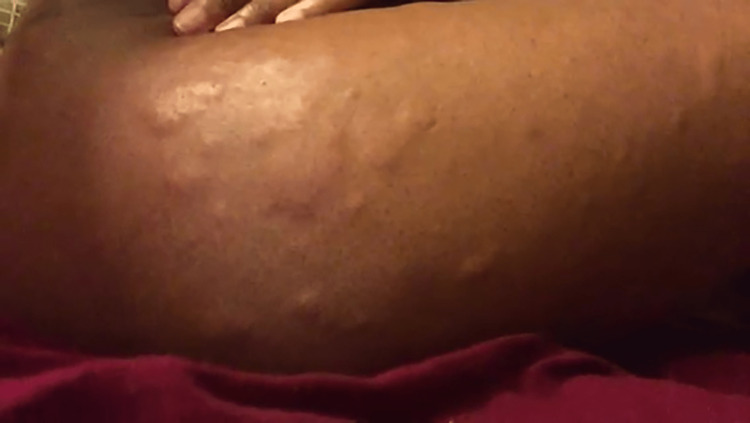
Urticaria involving the lateral aspect of the right thigh

We report the case of a 31-year-old female presenting with urticaria and angioedema, which occurred three to four days before her menstruation, extending two to three days after her menstruation began (Figures 1-2).

She reported swelling under her eyes and lips and an urticarial rash over her arms, back, buttocks, and thighs. She remained asymptomatic on the remaining days of the menstrual cycle. These episodes started after getting a medroxyprogesterone shot (104 mg) in 2016 for contraception. A day after this shot, she developed angioedema, profuse itching, and urticarial lesions all over her body, which she managed with antihistamines and oral steroids. Despite this history, the patient received another 104 mg medroxyprogesterone shot three months later, which resulted in a recurrence of urticaria and angioedema. She needed management with diphenhydramine, famotidine, and oral steroids, with some improvement in symptoms. She did not have any past medical problems. She did not have any history of allergies. She had no history of non-steroid anti-inflammatory drug use during menstrual cycles. She had an intrauterine device containing levonorgestrel inserted at the age of 20, which was removed after three to four years.

She became pregnant in January 2019, and her urticaria and hives became challenging to manage. She then had a spontaneous resolution of these skin changes in February and remained relatively symptom-free until her miscarriage in mid-March. She underwent dilation and curettage after the miscarriage, after which her symptoms returned immediately.

She continued to have symptoms around her menstrual cycles. She was then seen in an allergy and immunology clinic. She underwent a workup for hereditary angioedema and chronic spontaneous urticaria (Table 1).

**Table 1:** Laboratory evaluation of the patient WBC: white blood cell count; ESR: erythrocyte sediment rate

LABS	VALUE	NORMAL RANGE
Hemoglobin	12.3	11.1-15.9 g/dL
WBC	4600	3400-10800/uL
Platelet count	313,000	150000-379000/uL
Neutrophils	62	50-70%
Lymphocytes	28	18-42%
Monocytes	8	4-9%
Eosinophils	2	1-3%
Basophils	0	0-2%
ESR	16	<20 mm/hr
Total immunoglobulin E	4	6-495 IU/mL
Serum C1 esterase inhibitor	29	21-39 mg/dL
C1 esterase inhibitor function	>92	>67% mean normal

**Figure 3:**
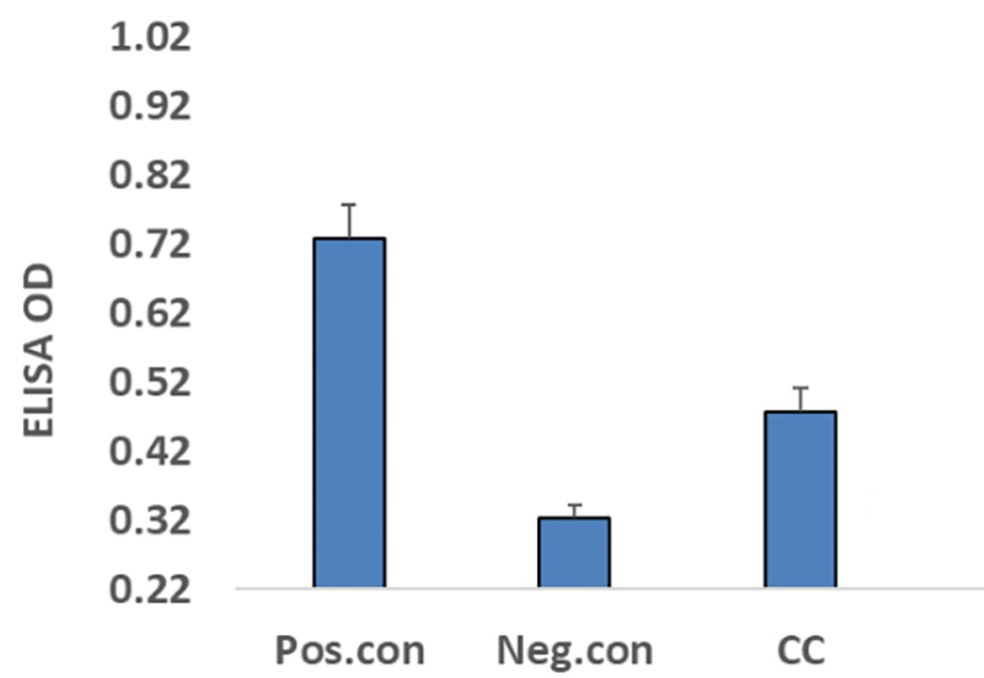
Results of serum progesterone-specific immunoglobulin E levels, determined using an enzyme-linked immunosorbent assay (ELISA) OD: optical density; Pos.con: positive control; Neg.con: negative con; CC: patient's initials

She was being managed with double the standard doses of second-generation antihistamines (including cetirizine, levocetirizine, fexofenadine, loratadine, and desloratadine) with partial relief. She even underwent treatment with montelukast (10 mg twice daily) and omalizumab, again with partial relief. Subsequently, due to the cyclic nature of her symptoms and her severe reaction to medroxyprogesterone, there was a concern for progesterone hypersensitivity. She underwent testing for progesterone-specific serum immunoglobulin E (IgE), which came back positive (Figure 3).

This test was performed using an IgE-specific enzyme-linked immunosorbent assay (ELISA). She did not undergo any skin testing.

She was then seen in an obstetrics and gynecology (OBGYN) clinic and started on cyclic monophasic oral contraceptive therapy to suppress ovulation. She had a complete resolution of symptoms for two to three years, after which the symptoms came back in a cyclical manner. She came to the endocrinology clinic for further management. She was recommended to initiate continuous oral contraceptive pill (OCP) use and skip the placebo pills to provide better control of her symptoms. She expressed her desire to become pregnant in the coming one to two years, so she was also referred to the allergy and immunology clinic for possible desensitization so that she could discontinue OCPs in the future and even avoid worsening symptoms during pregnancy.

## Discussion

We have presented the case of a 31-year-old female with progesterone hypersensitivity and a history of exogenous progesterone exposure. She presented with urticaria and angioedema, which only got partially relieved with antihistamines, montelukast, and omalizumab. Her symptoms were resolved by suppressing ovulation with monophasic cyclic oral contraceptive therapy for two to three years. She likely became re-sensitized to progesterone due to the cyclic use of OCPs. She then needed to switch to monophasic continuous OCP use.

Progesterone hypersensitivity is a rare disorder with cutaneous or systemic reactions to exogenous or endogenous progesterone. Most reported cases had prior exposure to an exogenous source [6]. The onset is at any time between menarche and menopause [7]. Presentations can vary from urticarial skin lesions, pruritis, angioedema, and asthma-like symptoms to severe ones like anaphylaxis and Steven Johnson syndrome [6,8-10].

The proposed mechanism behind PH is considered a hypersensitivity reaction, not an autoimmune process [1]. Some patients have presentations of immediate or type 1 hypersensitivity with symptoms related to mast-cell activation like urticaria, angioedema, and anaphylaxis [11]. Some patients have symptoms consistent with delayed or type 4 hypersensitivity [11].

Cyclical aggravation of symptoms is the most significant clue for diagnosis. The diagnosis can also be made using progesterone skin testing (testing type 1 and type 4 hypersensitivity reactions) or a serum-based progesterone-specific IgE immunoassay (testing type 1 hypersensitivity reactions) [12,13]. The sensitivity and specificity of these tests for the diagnosis of PH have yet to be confirmed and can yield equivocal results [4]. In cases of high clinical suspicion but negative skin or IgE testing, a trial of drugs (OCPs, gonadotrophin receptor (GnRH) agonists, tamoxifen) can be given for three months to confirm the diagnosis [11].

The goals of therapy need to be discussed with the patient and should be determined based on whether the patient desires to be pregnant in the near future. In women who do not want pregnancy, symptoms can be alleviated using antihistamines, topical, or oral glucocorticoids. Long-term control can be achieved with medications that suppress ovulation. These include oral contraceptive therapy, GnRH agonists, and selective estrogen receptor modifiers. Since OCPs contain progesterone, some patients may develop worsening symptoms, and this option cannot be chosen for patients who develop PH secondary to OCP exposure [14]. Patients who fail these strategies need slow or rapid desensitization with oral or intramuscular (IM) progesterone. Patients who are pregnant, undergoing fertility treatments, or who have severe manifestations like anaphylaxis will need rapid desensitization [6]. Omalizumab may be used in refractory cases and cases with anaphylaxis [15]. Bilateral oophorectomy provides definitive management in severe cases refractory to other treatments [16,17].

## Conclusions

Women in the childbearing age group presenting with cyclical cutaneous and respiratory systems in the luteal phase and improving a few days after the initiation of menstrual cycles should be evaluated for progesterone hypersensitivity. A timely diagnosis can lead to the institution of therapy based on the patient’s desire for pregnancy with care from a multidisciplinary treatment team, including an allergist and immunologist, a reproductive endocrinologist, and/or an OBGYN provider.
